# Paper-Based Microfluidics Platform for Enhanced On-Site Electrochemical Detection of Manganese in Water

**DOI:** 10.1021/acsomega.5c09688

**Published:** 2026-03-06

**Authors:** Enahoro Asein, Selina Kern, Alexander Iles, Cartl-Magnus Morth, Pablo Gimenez-Gomez, Nicole Pamme

**Affiliations:** † Department of Chemistry, 7675 Stockholm University, Stockholm SE-106 91, Sweden; ‡ Wallenberg Initiative Materials Science for Sustainability, Department of Chemistry, Stockholm University, Stockholm SE-106 91, Sweden; § Department of Geological Sciences, 7675 Stockholm University, Stockholm SE-106 91 Sweden

## Abstract

Manganese pollution in water has severe toxic effects on biological organisms, including humans. Early detection is crucial to apply timely corrective measures and prevent irreversible consequences. Standard monitoring methods are expensive, time-consuming, and difficult to implement in resource-limited settings, leading to inefficient pollution remediation. The low cost and small size of screen-printed electrodes make them well-suited for on-site analysis but require flow conditions for sensitive results, making them difficult to implement in real-world scenarios. To address this challenge, we present the use of paper microfluidics in combination with screen-printed carbon electrodes for the electrochemical detection of manganese in water. Capillary-driven flow using paper microfluidics provided a practical fluid delivery method for on-site electroanalysis, which was compared to conventional flow methods, i.e., batch setups and flow cells, to demonstrate fitness for purpose. The sensing platform showed a linear response to Mn­(II) up to 200 μg L^–1^, with a detection limit of 0.69 μg L^–1^, well below the WHO guideline value. The response of the sensor was also analyzed in the presence of potentially interfering metals, and validated in real samples, showing excellent agreement with a standard inductively coupled plasma optical emission spectroscopy method. This approach, inducing flow with paper, is more sustainable, simple, portable, and cost-effective compared to the conventional methods, resulting in a setup with high applicability toward the detection of other pollutants such as metals, PFAS, and pharmaceuticals.

## Graphical abstract

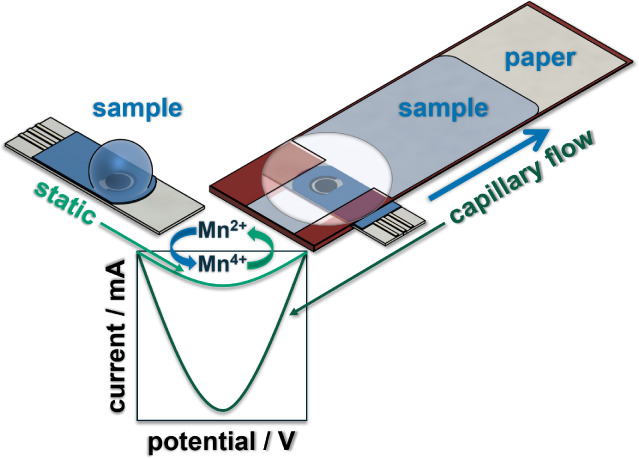

## Introduction

The monitoring of heavy metals in the environment is vital for safe-guarding the health of animals and plants.^1^ One of the most abundant is manganese,^2^ acting as a cofactor for photosynthesis in plants and enzymatic activity in animals.^3,4^ Manganese has 11 possible oxidation states ranging from −3 to +7 with five common states (+2, +3, +4, +6, +7); however, the +2 state is the dominant form present in the environment and biological fluids.^5,6^

In 2021, the World Health Organization (WHO) published a health-based guideline value of 80 μg L^–1^ for manganese in drinking water, particularly for bottle-fed infants but applicable to the general population.^7^ Anthropogenic activities such as mining, steel manufacturing, and battery production have heavily contributed to the accumulation of manganese in various bodies of water, surpassing the recommended levels by WHO in some areas around the world.^8^ At high levels, manganese has severe effects on the environment, humans, and other biological organisms.^8,9^ Manganese toxicity causes neurological deficiencies in humans^10^ and oral exposure through contaminated water sources can trigger cognitive and behavioral issues in children.^11–15^ Therefore, accurate and usable systems need to be developed to monitor and control the levels of manganese in drinking water.

The standard analytical methods for monitoring manganese concentrations in water use spectroscopy and spectrometry techniques, i.e., graphite furnace atomic absorption spectroscopy, inductively coupled plasma optical emission spectroscopy (ICP-OES), and inductively coupled plasma mass spectrometry.^16^ Although they allow for the detection of manganese at very low levels with high sensitivities and specificities,^17^ analyses take a long time and are expensive because they require bulky instrumentation operated by experts in centralized laboratories, limiting their applicability for frequent environmental monitoring. These are major drawbacks for monitoring heavy metals, especially in resource-limited settings where fast, cheap, and portable analysis is necessary.

Some portable analyzers have been proposed, mainly based on colorimetric^18,19^ or electrochemical^20–22^ detection. Electrochemical methods outperform colorimetric ones in terms of detection limits for heavy metals, with stripping voltammetry being the most used electroanalytical technique to detect manganese at low concentrations.^23–35^ This technique involves a preconcentration step, where the dissolved target analyte is deposited onto the surface of the working electrode, and then a stripping step is applied to remove the deposited form from the electrode. The current measured during stripping is proportional to the amount of analyte deposited during the preconcentration step, which is in turn proportional to the concentration of the analyte in the solution.^36^ Of the different variations, cathodic stripping voltammetry (CSV) offers the greatest advantages for manganese detection in terms of linear range, detection limit, analytical sensitivity, and insensitivity to oxygen when compared to anodic stripping voltammetry.^29^,,^31–33^

In CSV, the analyte is deposited by oxidation during the preconcentration step and stripped by reduction. Dynamic, flow-induced, sample conditions used during the preconcentration step improve the analytical performance of stripping voltammetry methods by facilitating greater mass transport of the analyte during preconcentration.^37^ It is conventionally done by magnetic stirring in a batch setup or by flow injection. However, batch and flow injection setups require additional instrumentation, i.e., stirrers and pumps, limiting their use for on-site environmental monitoring. As an alternative, the porous nature of paper which allows for liquid wicking by capillary action could be used to create dynamic conditions by providing a continuous flow of sample through the paper. A direct contact between the paper and electrodes can enhance preconcentration during stripping voltammetry, as has been reported in the literature.^38–40^ Though, to the best of our knowledge, no one has applied it toward manganese analysis or compared the performance of paper with conventional and well-defined flow setups.

Here, a method that combines the use of a commercial screen-printed carbon electrode (SPCE) with the capillary-driven flow of a paper device for the detection of Mn­(II) by cathodic stripping square wave voltammetry is presented. The method based on capillary-induced flow using paper was combined with a portable potentiostat to demonstrate its accessibility for on-site analysis. We report the selection of electroanalytical conditions, i.e., pH, deposition and stripping potentials, and deposition time during CSV. The analytical performance of the device was evaluated in terms of sensitivity, limits of detection and quantification, and selectivity. Furthermore, the analytical performance of the paper-based setup was compared to the performances of conventional batch and flow setups, to demonstrate its real impact. Finally, the paper-based platform was further validated by analyzing real water samples, tap water and groundwater from a mine, and comparing the results to those obtained using a gold-standard method, ICP-OES.

## Experimental Section

### Reagents and Solutions

Potassium acetate was purchased from Thermo Fisher Scientific (Stockholm, Sweden). Manganese standard solution (1000 mg L^–1^, Mn­(NO_3_)_2_ in 0.5 M HNO_3_), ultrapure nitric acid (65%), iron­(II) chloride tetrahydrate (≥99.0%), copper­(II) sulfate pentahydrate (≥98.0%), and lead nitrate (≥99.0%) were purchased from Sigma-Aldrich (Stockholm, Sweden). Hydrochloric acid (37%) was purchased from VWR (Stockholm, Sweden). Acetic acid (≥99.8%) and sodium hydroxide (99–100.5%) were purchased from Honeywell (Stockholm, Sweden). Deionized water from a Milli-Q IQ 7000 system (resistivity >18.2 MΩ·cm, Merck, Stockholm, Sweden) was used to prepare all aqueous solutions.

### Equipment

The paper device design was printed using a ColorQube 8570 (Xerox, Connecticut, USA) printer loaded with wax cartridges. A laminator (Saturn 3i, Fellowes Brand, Illinois, USA) was used to melt the wax printed on the paper surface. The stirred conditions for the batch setup were created using a Variomag mono magnetic stirrer (H+P Labortechnik, Schwerte, Germany). The flow cell was fabricated with an M8CUBE Computer Numerical Control (CNC) drilling machine from Datron (New Hampshire, USA), and the liquid flow was controlled with a Pump 11 Elite syringe pump from Harvard Apparatus (Massachusetts, USA). pH was measured using a model 924 005 glass pH electrode and a model 3510 digital pH meter (Jenway, Cole-Parmer, Illinois, USA). The electrochemical measurements were performed with a potentiostat, PalmSens4 and SCPEs, DRP-110 from Methrohm Dropsens (Asturias, Spain). The ICP-OES measurements were performed using an iCAP 6500 DUO from Thermo Fisher Scientific at the Stable Isotope Lab, Department of Geological Sciences, Stockholm University.

### Design, Fabrication, and Operation of the Paper-SPCE Sensing Platform

The paper device (26 mm wide and 120 mm long) was designed with AutoCAD (Autodesk, California, USA) and printed onto Whatman grade 4 qualitative filter paper (205 μm thickness, 20–25 μm particle retention, 92 g m^–2^ basis weight, ≤0.06% ash content, Cytiva, Uppsala, Sweden) using the Xerox printer. Whatman 4 filter paper was selected due to its higher porosity, compared to the other offerings from Whatman,^41^ leading to a faster flow rate and an enhanced mass transport to improve the electroanalysis performance. The device had two wax-free hydrophilic areas (Figure 1a, from left to right, in gray color): (i) a 12 mm wide and 30 mm long channel to control the liquid flow over the electrode surface; and (ii) a 26 mm wide and 59 mm long reservoir area for increased liquid flow through the channel. Wax (red color in Figure 1a) printed onto the top of the paper was melted through the paper using the laminator at 125 °C (Figure S1, in the Supporting Information), creating a hydrophobic barrier that limited liquid movement to the hydrophilic channel and reservoir areas. A holding area of 26 mm width and 10 mm length at the bottom of the hydrophilic channel was defined to place the back side of the SPCE with double-sided tape (GPT-020F, 3M, Stockholm, Sweden). Then, the paper device was folded with the electrode surface in contact with the hydrophilic channel (Figure 1b). As a proof of concept, a paper clip was used to hold the SPCE and the paper device together, ensuring sufficient contact between the hydrophilic channel and the electrode surface. To create the dynamic sample conditions during analysis, the paper-SPCE sensor was dipped vertically into a Petri dish containing the sample (Figure 1c). The electrochemical measurement, i.e., deposition, was started immediately after the liquid front passed the SPCE surface (5 s after dipping the paper in the sample). The paper device was designed to match the wetting time of the hydrophilic areas to the selected deposition time of 600 s.

**Figure 1:**
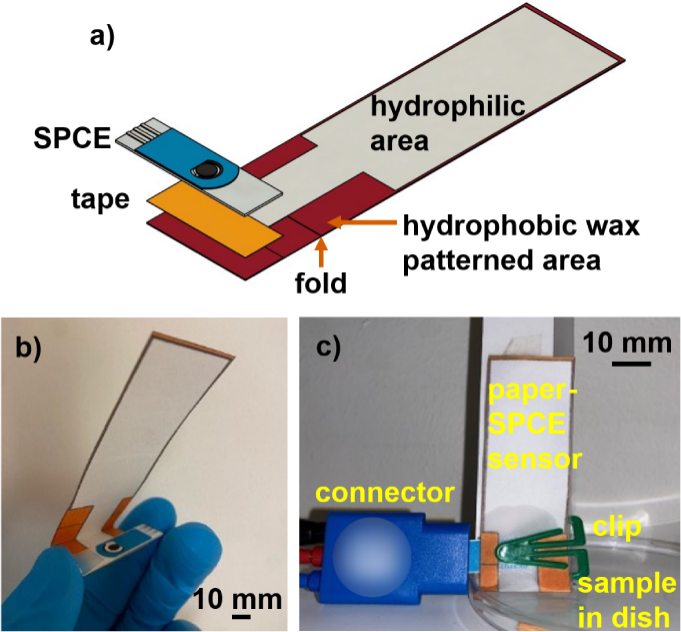
Paper-SPCE sensing platform used for Mn­(II) detection. (a) Schematic of the sensing platform. (b) Photograph of the fabricated paper device combined with a commercial SPCE. (c) Photograph of the platform during operation.

### Liquid Flow through the Paper Device

The average flow rate of water through the paper device was determined by allowing liquid to flow through the paper-SPCE sensor for 10 min. The difference in the mass of water in the Petri dish before and after wicking was used to calculate the volume of water traveling through the paper device. This was in turn used to calculate the average flow rate through the paper device.

The flow profile for the paper-SPCE sensor was obtained by recording the time required for water (blue food dye added for visibility) to travel intervals of 5.4 mm from the bottom to the top of the reservoir area.

### Batch Setup

The batch setup consisted of a 50 mL beaker placed on the magnetic stirrer with a magnetic stir bar (Figure S2, in the Supporting Information). Measurements were done either under unstirred (0 rpm) or stirred conditions (1000 rpm).

### Design and Fabrication of the Flow Cell

The microfluidic flow cell, made of poly­(methyl methacrylate) (PMMA), was designed with AutoCAD and fabricated via CNC machine milling. The flow cell (30 mm wide and 35 mm long) was formed by two layers of PMMA (4 mm and 9 mm thick top and bottom layers, respectively) held together with screws (Figure S3a, in the Supporting Information). The bottom layer contained a 10.2 mm wide, 28 mm long, and 0.5 mm deep area to house the SPCE. The top layer contained a 7.5 mm wide, 16 mm long, and 0.2 mm deep elongated hexagonal area with two 1 mm diameter holes, serving as inlet and outlet to move liquid over the electrode surface. A syringe pump was used to introduce solutions into the microfluidic flow cell (Figure S3b, in the Supporting Information). Liquid was pumped into the flow cell at a flow rate of either 1 mL min^–1^ or 40 μL min^–1^.

### Electroanalytical Conditions

CSV was used as the electroanalytical technique for quantification (Figure S4a, in the Supporting Information). Square wave, used in this study, is one of three common waveforms used during the stripping step, the other two being linear sweep and differential pulse.^42^ The combined square wave and staircase potential waveforms (Figure S4b, in the Supporting Information) improves analytical sensitivity by minimizing the capacitive current.^43^

First, the effect of pH on Mn­(II) detection was assessed by cyclic voltammetry (CV) at 0.1 V s^–1^ as the scan rate and 10 mV as the potential step, in a potential range from −0.2 to 1.0 V. The experiments were performed using the unstirred batch setup, in buffer solutions containing 10 mg L^–1^ of Mn­(II) with the pH adjusted between 2.5 to 11.8.

Then, the applied potential during the preconcentration step, 0.9 V, and the potential range, 0.8 to 0 V, during the stripping step of square wave voltammetry (SWV) experiments were selected from the cyclic voltammograms (CVs). The amplitude, potential step, and frequency were set at 25 mV, 4 mV, and 14.24 Hz, respectively. Different time durations, in the range of 2.5 to 20 min, were tested for the preconcentration step using the stirred batch setup.

The performances of the different measurement setups were compared, using both CV and SWV, to study the suitability of the developed paper-based sensing platform for manganese detection. CV measurements were carried out using a 10 mg L^–1^ Mn­(II) solution. All CV measurements were done under static conditions. Static conditions were achieved by not stirring the batch setup and by filling up the area above the SPCE in the flow cell then turning off the syringe pump. For the paper-based setup, static conditions were achieved by recording the CVs after complete wetting of the hydrophilic areas. Square wave voltammograms (SWVs) were obtained from Mn­(II) standard solutions in the range of 0 μg L^–1^ to 200 μg L^–1^. Flow conditions were only used during the preconcentration step. Electrochemical impedance spectroscopy (EIS) diagrams obtained at 0.1 V excitation amplitude in the 100 kHz to 100 mHz frequency range were used to compare the solution resistance across the different setups.

All measurements were carried out in 0.1 M potassium acetate buffer. The reported potential values are all against the Ag/AgCl pseudoreference electrode. Before use, SPCEs were electrochemically cleaned by performing one SWV measurement in a 0.1 M acetate solution using the unstirred batch setup.

### Study of Potentially Interfering Ions

The effects of three cations, Cu­(II), Fe­(II), and Pb­(II), on the CSV method using the paper-SPCE sensing platform for Mn­(II) detection were evaluated. Standard solutions containing the interfering ions at a concentration of 100 μg L^–1^ were analyzed in the presence and absence of 100 μg L^–1^ Mn­(II). Further experiments to understand the behavior of interfering ions were done by CV using standard solutions containing the ions at a concentration of 10 mg L^–1^.

### Analysis of Real Water Samples

Three real water samples were used to validate the performance of the developed sensing platform. The first sample was tap water spiked with 70 μg L^–1^ Mn­(II) using the manganese standard solution. The two other samples were environmental samples collected from the top of a rock wall at the Ytterby mine, Resarö, Sweden (59°42′84“N 18°35′38”E). The manganese concentration in the Ytterby mine tunnels plays a key role for mapping microorganisms and element distribution, helping to understand the factors driving these microbial communities and mineral deposits. The mine samples were collected at different times and stored at 4 °C until analysis. All three samples were analyzed with the paper-SPCE sensing platform and by ICP-OES. Samples analyzed with the paper-SPCE platform were diluted in 0.3 M acetate buffer using a 2:1 sample to buffer ratio. Nitric acid (1% of sample volume) was added to samples for ICP-OES analysis to ensure the stability of metals present in the sample.

## Results and Discussion

### Selection of the Parameters for Manganese Analysis

The performance of electroanalysis depends on the pH; therefore, the optimal pH was first studied and results presented in Figure 2. At pH 2.5 (blue colored line) and pH 11 (pink colored line), no oxidation or reduction peak for Mn was observed. The absence of peaks at pH 2.5 can be attributed to the oxygen reduction reaction (ORR) occurring at a similar potential as the Mn^2+^ ⇌ MnO_2_ redox reaction. This is supported by the Pourbaix diagram of manganese^44^ which shows the ORR potential of water close to the Mn^2+^ ⇌ MnO_2_ redox potential. In the case of pH 11, a brown precipitate was observed after preparing the solution (Figure S5, in the Supporting Information), resulting from a rapid atmospheric oxidation of Mn­(OH)_2_ to MnO­(OH);^45^ hence, no peaks could be observed in the CV. At pH 4.8 (orange colored line) and 5.8 (green colored line), the expected oxidation/reduction peaks for the Mn­(II)/Mn­(IV) redox pair were observed. The shift between the peaks could also be explained using the Pourbaix diagram which shows a descending slope for the Mn^2+^ ⇌ MnO_2_ equilibrium line, i.e., the redox potential is inversely proportional to the pH. The effect of pH on the CVs for the Mn^2+^ ⇌ MnO_2_ redox reaction we observed are in line with those reported by previous works.^28,46^ The same solutions of 0.1 M acetate buffer without manganese were analyzed by CV (Figure S6, in the Supporting Information) and no peaks were observed, demonstrating that all previously observed peaks were due to Mn^2+^ ⇌ MnO_2_ redox processes. From the results, pH 4.8 was selected as optimal for the next experiments because it showed distinct peaks.

**Figure 2:**
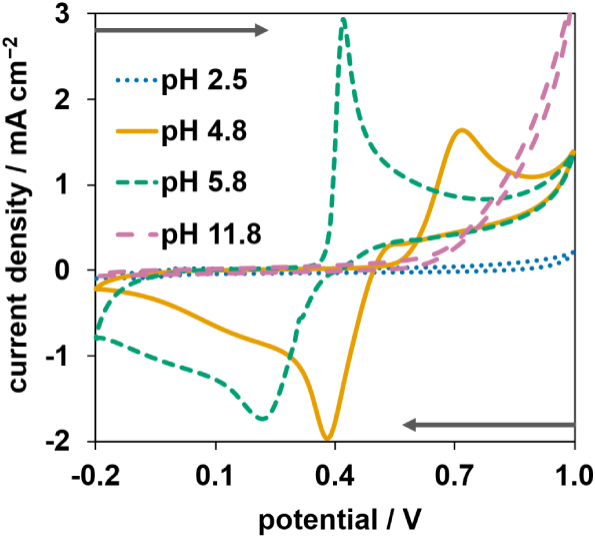
Cyclic voltammograms recorded at 0.1 V s^–1^ from −0.2 to 1.0 V (vs Ag/AgCl pseudoreference electrode) in a 0.1 M potassium acetate solution containing 10 mg L^–1^ Mn­(II), at pHs of 2.5 (blue), 4.8 (orange), 5.8 (green), and 11.8 (pink). The gray arrows indicate the directions of the measurements. Measurements were done using the unstirred batch setup.

The deposition potential and stripping range for SWV were selected from the CV obtained at pH 4.8 (orange colored line in Figure 2). A deposition potential of 0.9 V, well past the oxidation peak, was chosen to guarantee the complete oxidation of Mn­(II) to Mn­(IV). For the stripping step, a range from 0.8 to 0 V was selected because it covered the entire width of the reduction peak. The parameters for the square wave waveform were set similar to those used by Kang et al.^21^ The effect of the deposition time on the SWV signal was evaluated in 100 μg L^–1^ Mn­(II) solutions using a stirred batch setup with different deposition times in the range of 150 to 1200 s. Figure S7, in the Supporting Information shows proportionally increasing peak areas up to 1200 s, as expected, because a longer deposition time allows for greater current generation during the stripping step. However, 600 s was selected for further measurements because it offered a good balance between sensitivity and analysis time.

### Comparative Study of the Measurement Setups

To study the suitability of the paper-based setup for manganese sensing, the different measurement setups were compared using CV and SWV techniques. All CV measurements were performed under static conditions to ensure that the electron transfer at the electrode surface was strictly limited by diffusion, allowing for a better comparison of the obtained CVs. No peaks were observed for solvent blank measurements using the different setups (Figure S8a, in the Supporting Information). After adding Mn­(II) to the buffer solutions, the three setups showed similar oxidation and reduction peaks from CV experiments (Figure S8b, in the Supporting Information), demonstrating their suitability for monitoring the Mn^2+^ ⇌ MnO_2_ redox process. The slightly lower peak currents observed with the paper-based setup was hypothesized to be due to higher resistance from the reduced effective contact area between the electrode and the sample flowing through the paper matrix. It was confirmed by EIS (Figure S9, in the Supporting Information), that the displaced real part of the impedance indicated a higher solution resistance for the paper-based setup compared to the batch and flow cell setups. Nevertheless, the contact between the wetted paper and the SPCE proved to be sufficient for further electroanalysis.

The analytical performance of the measurement setups for the quantification of Mn­(II) by CSV, i.e., sensitivity, limit of detection (LoD), and limit of quantification (LoQ), were evaluated and compared (Figure 3a–e). The flow conditions had an effect on the SWV signal, with a significant difference in the recorded current for the setups with dynamic conditions compared to the unstirred batch setup. This is due to the well-known fact that the amount of analyte deposited during the preconcentration step depends on the electron transfer rate at the electrode surface and the mass transfer rate of fresh analyte to the surface.^47^ In most cases, and particularly in diffusion-controlled setups, the mass transfer rate is the limiting step; consequently, replenishment of the analyte at the electrode surface is crucial for greater deposition. The magnetic stir bar rotating at a speed of 1000 rpm provided visibly faster liquid flow to the electrode surface compared to other setups, resulting in the highest recorded signal. The flow cell setup operated at a 1 mL min^–1^ showed the second highest signal; however, when the flow rate was reduced to 40 μL min^–1^ to match the average flow rate through the paper device, (37 ± 4) μL min^–1^, the recorded current decreased to similar values as the paper-based one. This indicates that the sensitivity of the measurements with the paper-based setup is primarily limited by the flow rate of liquid through the device, which could be improved by selecting a different paper substrate with higher porosity and faster liquid wicking rate. Additionally, the Whatman 4 filter paper used in this work contributed to the electroanalytical signal, as evidenced by the higher peak currents observed in the 0.1 M acetate blank SWV measurements (Figure S10a, in the Supporting Information). This was further confirmed by adding pieces of filter paper to 0.1 M acetate buffer and performing the SWV measurement with the stirred batch setup (i.e., 50 mL beaker placed on a magnetic stirrer with a magnetic stir bar at 1000 rpm during the deposition and stopping the stir bar for the SWVs). The ratio of paper in the 12 mm × 30 mm hydrophilic channel of the paper-SPCE sensor to the total volume of liquid flowing through the paper device (370 mm^3^) was used for the measurement in the stirred batch setup. Figure S10b, in the Supporting Information, shows that the presence of paper in the acetate buffer results in a signal with a second peak appearing between 0 and 0.3 V. Further research to analyze if species leach out of the paper is needed. Moreover, the use of different paper substrates could result in different signal contributions.^48^

**Figure 3:**
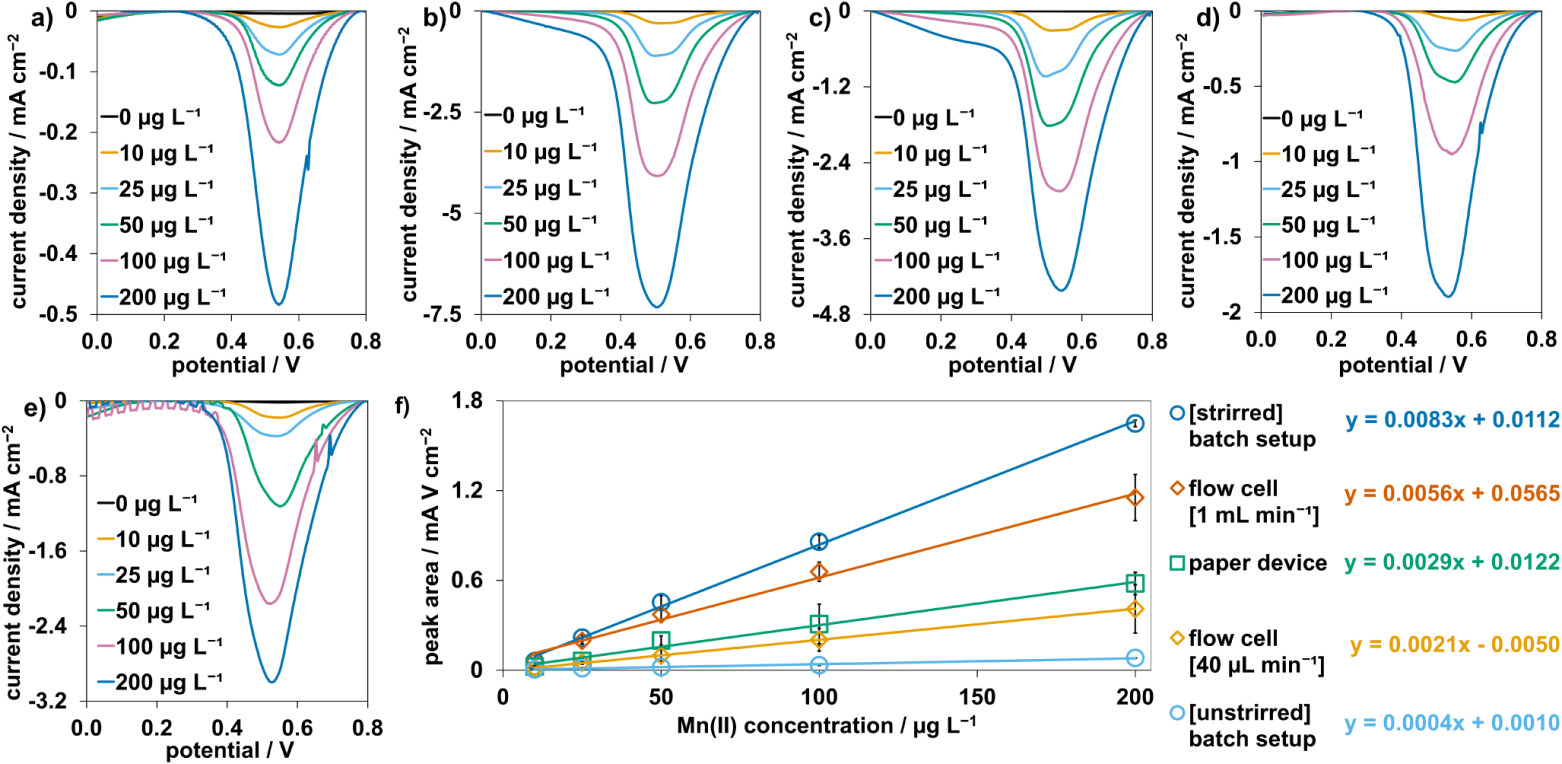
(a–e) Square wave voltammograms (0.9 V deposition potential, stripping range from 0.8 to 0 V with 25 mV amplitude, 4 mV potential step, and 14.24 Hz frequency (vs Ag/AgCl pseudoreference electrode)) recorded for a 0.1 M potassium acetate solution containing 0 μg L^–1^ to 200 μg L^–1^ Mn­(II) using the different measurement setups. (a) Batch setup without stirring. (b) Batch setup with stirring at 1000 rpm using a magnetic stir bar. (c) Flow cell setup with a 1 mL min^–1^ flow rate. (d) Flow cell setup with a 40 μL min^–1^ flow rate to match the average flow rate through the paper device. (e) Paper-based setup. (f) Calibration curves obtained using the peak areas from measurements done with the unstirred batch setup (light blue), the stirred batch setup (dark blue), the flow cell at 1 mL min^–1^ (dark orange), the flow cell at 40 mL min^–1^ (light orange), and the paper device (green) in the range of 0 μg L^–1^ to 200 μg L^–1^ Mn­(II). Each point is the mean peak area of three replicates, with error bars representing standard deviations.

The area under the peaks in the studied potential range was used as the analytical signal to obtain the calibration curves shown in Figure 3f. A linear response was obtained in the concentration range of 10 μg L^–1^ to 200 μg L^–1^ of Mn­(II) for all the setups, with a similar sensitivity for the paper-based and the flow cell setups running at a similar flow rate, 40 mL min^–1^. The LoD and LoQ for all the setups were calculated using the formula 3.3σ/slope and 10σ/slope, respectively, where σ is the standard deviation of the blank (n = 3).^49^ The calculated LoDs and LoQs, presented in Table 1, demonstrate the need for higher sensitivities to reach lower detection and quantification limits. The paper-based setup with an LoD of 0.69 μg L^–1^ and LoQ of 2.1 μg L^–1^ displays excellent suitability for the analysis of manganese concentrations well below the WHO guideline value of 80 μg L^–1^.^7^

**Table 1:** Summary of Analytical Performance Parameters Calculated for the Different Measurement Setups

Setup	LoD/μg L^–1^	LoQ/μg L^–1^
[Stirred] batch setup	0.054	0.16
Flow cell [1 mL min^–1^]	0.32	0.96
Paper-based	0.69	2.1
[Unstirred] batch setup	1.3	3.8
Flow cell [40 μL min^–1^]	2.5	7.6

### Evaluation of Liquid Flow through the Paper Device

Unlike the batch and flow setups where flow rates remain constant throughout the preconcentration step, the nature of capillary action through the paper device results in varying flow rates. The Lucas–Washburn equation, which relates the distance traveled by a liquid to time, has been used to model capillary flow in vertical porous systems.^50^ Although, the equation fails when applied outside its boundary conditions, e.g., non-negligible gravity regime, heterogeneous porous media, and nonuniform geometries, the underlying concept—the distance traveled versus time, linear velocity, approaches a limiting value—still holds. The nonconstant linear velocity for liquid traveling through our paper device is shown in Figure S11, in the Supporting Information. The average linear velocity during the first 10 mm of travel was more than 3× that of the final 10 mm. The flow profile of liquid through our paper device demonstrated a logarithmic decay. This presents an opportunity for future research to explore the impact of various geometries and device design on the flow profile and sensitivity of stripping voltammetry methods using paper for liquid flow.

### Interference Study for the Paper-SPCE Sensor

The selectivity of the sensor was tested in the presence of three different metal ions, Cu­(II), Fe­(II), and Pb­(II), which were selected because their Pourbaix diagrams show redox processes occurring at around the same potential as the Mn^2+^ ⇌ MnO_2_ redox process.^44^ As­(III), another metal ion with redox processes around that potential, was not evaluated due to its unlikely presence in drinking water sources. The +2 oxidation state was selected for examination as it is the most soluble form of the metals in water. Measurements were done with the SWV method, using the paper-SPCE sensor, with a concentration of 100 μg L^–1^ for all ions. Interferents by themselves did not give a significant signal (orange bars in Figure 4a); however, their effect was prominent when present with Mn­(II) ions at the same concentration. The height of the blue bars in Figure 4a show that the highest interference was observed when all three metal ions were present, with Cu­(II) exhibiting the greatest individual interference to the peak area. Fe­(II) had the lowest interference on the peak area, although a significantly lower peak height for Fe­(II) due to peak broadening was observed (Figure 4b). These interferents are typically not present in drinking water at the high concentrations evaluated in this study; however, care should be taken when analyzing more complex samples.

**Figure 4:**
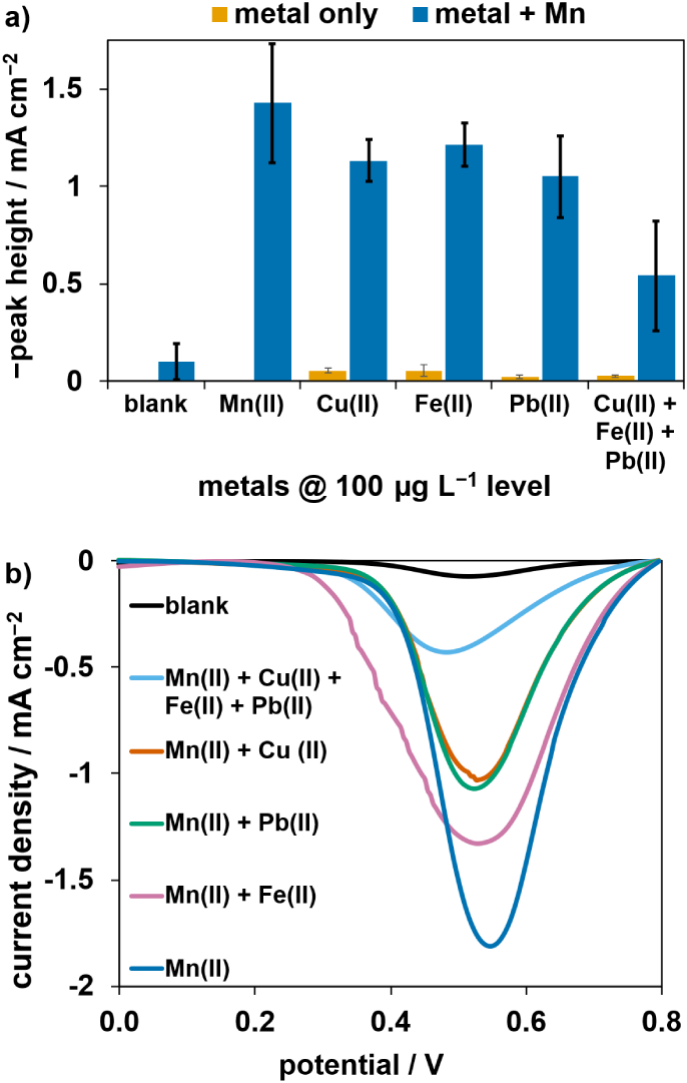
Effect of interferents on Mn­(II) analysis with the paper-SPCE sensor. (a) Solutions containing 100 μg L^–1^ of the metal ions were measured in the absence (orange bars) and presence (blue bars) of 100 μg L^–1^ Mn­(II). The error bars represent standard deviations obtained from three replicates. (b) Square wave voltammograms obtained from the analysis of 100 μg L^–1^ Mn­(II) and 100 μg L^–1^ of the interferents.

Cyclic voltammetry experiments were performed using the unstirred batch setup to verify the interfering effect of the selected cations on the Mn^2+^ ⇌ MnO_2_ redox process. In the first instance, measurements of the interferents at 10 mg L^–1^ were performed (Figure S12a, in the Supporting Information). Only Fe­(II) showed a clear redox response in the tested −0.2 to 1.0 V range. When both the interferents and Mn­(II) were present at 10 mg L^–1^, the CVs obtained in the presence of Fe­(II) and Pb­(II) differed from the pure Mn­(II) one (Figure S12b, in the Supporting Information). The interferences observed in this study are in line with the results from Berg et al., who also measured Mn­(II) concentrations by square wave CSV on a carbon electrode.^33^ An explanation for this behavior could be that Fe­(II) and Pb­(II) have higher oxidation states of +3 and +4, respectively, which allow for oxidation and subsequent reduction, contributing to their influence on the Mn­(II) CV in Figure S12b, in the Supporting Information. Lima et al. proposed the codeposition of Pb­(II) as PbO_2_ on the electrode as the cause for inference in Mn­(II) detection.^32^ This same argument could be applied to the codeposition of Fe­(II) as Fe_2_O_3_. The absence of significant peaks in the SWVs for Fe­(II) and Pb­(II) may be due to optimization of the stripping range for Mn­(II) and/or inherent difficulty in stripping their respective oxides. The interference from Cu­(II) presents a challenging case against the proposed mechanism of codeposition as the deposition of a higher oxidation state is very unlikely. The lack of clear explanations for the interferences in this work and in the literature indicates the need for future work to explore the theory behind them and efforts to mask the most significant ones.

### Real Sample Analysis

The use of the developed paper-SPCE sensor for monitoring Mn­(II) was validated in real water matrices. The results in Table 2 demonstrate a very good agreement between the sensor and the ICP-OES method, particularly for the spiked tap water sample. Measurements of the nonspiked tap water sample showed no significant difference from a pure 0.1 M acetate blank and the certificate of quality from Stockholm Vatten och Avfall (Stockholm’s municipal water and waste management company), reported a value of <0.001 mg L^–1^ for manganese.^51^ As other components of drinking water were also reported to be at normal levels, the sample was considered ideal for spiking with Mn­(II). The low relative error of 2% demonstrated excellent recovery using the developed paper-SPCE sensor.

**Table 2:** Mn Concentrations Obtained from the Measurement of Real Samples^a^

	Mn concentration/μg L^–1^		
Sample	Paper-SPCE sensor	ICP-OES	Relative error
Spiked tap water	73 ± 10	71.4	+2%
Mine sample 1	89 ± 12	113.4	–21%
Mine sample 2	82 ± 14	102.7	–20%

a The errors represent standard deviations obtained from measurements with three different paper-SPCE sensors

On the other hand, samples collected from the top of a rock wall at the Ytterby mine were more complex with a high concentration of naturally occurring Mn.^52^ The concurrent presence of other elements at high concentrations increased the likelihood for interference; notwithstanding, the obtained relative error was below 21% for both samples, demonstrating the applicability of the proposed sensing platform for complex samples.

### Comparison with Other Methods for Detecting Manganese

The paper-SPCE sensing platform developed in this work was compared to other sensors proposed for the environmental monitoring of manganese in the literature (Table 3). The paper-SPCE sensor fits in with the trend of electrochemical methods outperforming optical methods. Importantly, it stands out among the electrochemical sensors in terms of its simplicity. Our sensor was able to reach detection limits comparable to the other electrochemical sensors without requiring an externally powered vibrating disc^22^ or stirrer^53^ for dynamic sample conditions. The absence of external power reduces the operational costs and makes the sensor well suited for use in on-site monitoring. Access to safe drinking water has been identified as a human right;^54^ hence, accessibility should play a major role in the design of sensors for water monitoring. Additionally, the sensing platform proposed here has a simple fabrication method which allows for mass production and easy operation. The flexibility of electroanalysis also makes our paper-based platform transferable to the monitoring of other contaminants.

**Table 3:** Comparative Analysis of Our Paper-SPCE Sensor with Other Manganese Sensors

Detection Method	Dynamic Condition	LoD / μg L^–1^	Reference
Colorimetric	n/a	110	^18^
Colorimetric	n/a	15	^19^
Optical	n/a	4	^55^
Electrochemical	Vibrating motor	0.56	^22^
Electrochemical	Magnetic stirring	0.76	^53^
Electrochemical	Paper wicking	0.69	This work

## Conclusions

In this study, an electroanalytical sensing platform for the detection of Mn­(II) in water by square wave CSV was developed. The platform uses the capillary action of a paper device to improve performance by providing continuous liquid flow to the surface of the electrode during preconcentration. The sensing platform has a low LoD of 0.69 μg L^–1^, making it well suited for Mn­(II) monitoring at a health-relevant level. The analytical performance of the paper-based platform was compared to conventional batch and flow cell setups used for electroanalysis. The use of paper microfluidics significantly improved analytical sensitivity with a 7-fold increase compared to a static measurement setup; the best flow setup in this study, using a magnetic stirrer, showed a comparatively smaller improvement in sensitivity over the paper-based one. The sensing platform showed good precision and accuracy in the analysis of real-world water samples with a 2% relative error for a spiked tap water sample and relative errors below 21% for more complex mine samples, compared against an ICP-OES method. These results demonstrate the high specificity of the developed platform for real applications.

This work shows that paper is a simple, sustainable, and suitable material for improving on-site environmental electroanalysis. Further work will focus on exploring different types of paper with faster flow rates and evaluating possible interferents. The paper-SPCE design can improve sensors for the electrochemical detection of many other analytes such as heavy metals, pharmaceuticals, PFAS, and more.

## Supplementary Material



## Notes

Sensor design workflow; batch and flow cell setups; CVs of blanks; impact of high pH on Mn­(II) stability; effects of deposition time on peak area; CVs, EIS spectra, and SWVs of different measurement setups; influence of paper on the analytical signal; effect of interferents on peak height; and CVs of interferents (PDF)

## #

SK: Friedrich-Alexander-Universität Erlangen-Nürnberg, 91058 Erlangen, Germany

## Abbreviations

CNC: computer numerical control
CSV: cathodic stripping voltammetry
CV: cyclic voltammetry/cyclic voltammogram
EIS: electrochemical impedance spectroscopy
ICP-OES: inductively coupled plasma optical emission spectroscopy
LoD: limit of detection
LoQ: limit of quantification
PMMA: poly­(methyl methacrylate)
SPCE: screen-printed carbon electrode
SWV: square wave voltammetry/square wave voltammogram
WHO: World Health Organization
