# Supplementary material for: Paper-Based Microfluidics Platform for Enhanced On-Site Electrochemical Detection of Manganese in Water
Source: ACS Omega. 2026 Mar 6;11(10):15933–41. doi: 10.1021/acsomega.5c09688 (PMC13000773; doi:10.1021/acsomega.5c09688)
Supplement: Supplementary file 1 [file ao5c09688_si_001.pdf]

Paper-Based Microfluidics Platform for Enhanced On-site Electrochemical Detection of Manganese in Water

Enahoro Asein[1,2], Selina Kern[1], Alexander Iles[1], Cartl-Magnus Morth[3], Pablo Gimenez-Gomez[1*], Nicole Pamme[1,2]*

[1] Department of Chemistry, Stockholm University, SE-106 91 Stockholm, Sweden

[2] Wallenberg Initiative Materials Science for Sustainability, Department of Chemistry, Stockholm University, SE-106 91 Stockholm, Sweden

[3] Department of Geological Sciences, Stockholm University, SE-106 91 Stockholm, Sweden

* Corresponding author: pablo.gimenez-gomez@su.se (P.G.G.); nicole.pamme@su.se (N.P.)

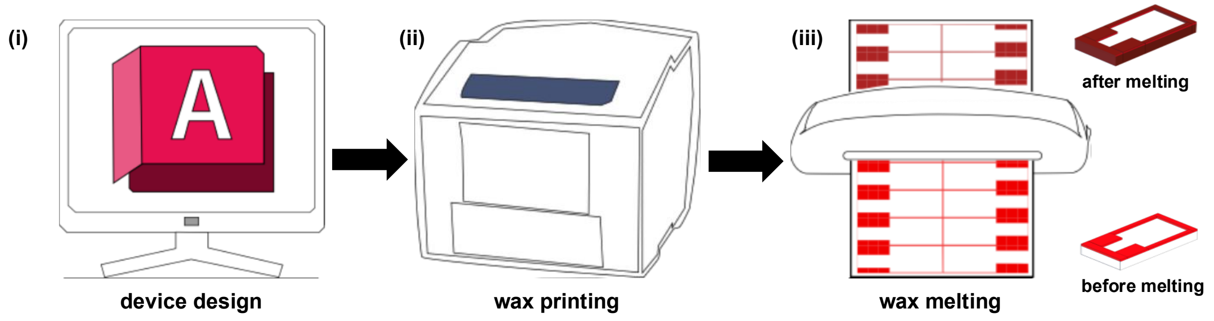

Figure S1. Workflow for paper device fabrication. **(i)** Design of the device in AutoCAD. **(ii)** Printing of the device on filter paper with a wax printer. **(iii)** Melting of the wax printed layer through the paper using a laminator.

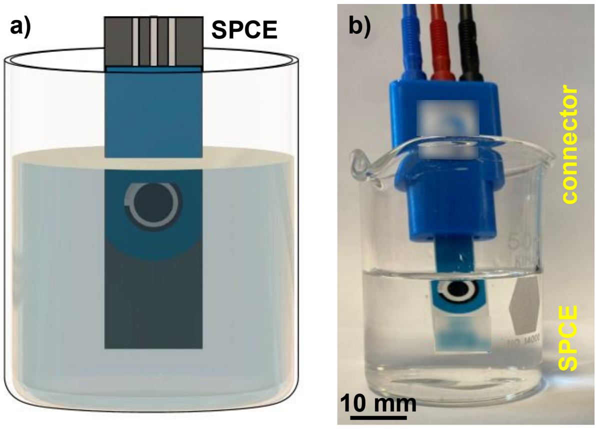

Figure S2. Batch setup. **(a)** Schematic representation of a SPCE dipped into the sample. **(b)** Photograph of the batch setup during operation.

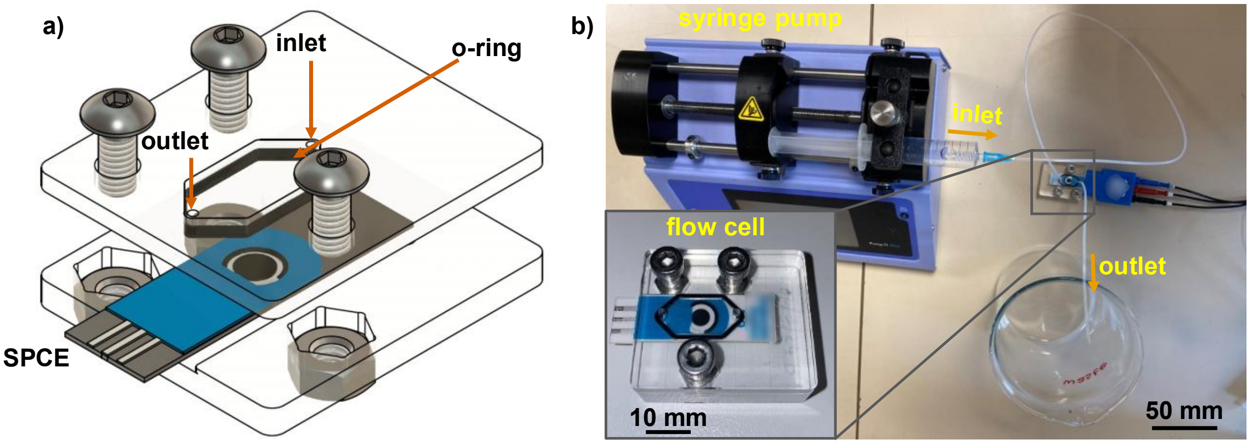

Figure S3. Flow cell setup. **(a)** Schematic representation of the microfluidic flow cell used for measurements. **(b)** Photograph of the setup during operation with a syringe pump pushing liquid through the flow cell and over the SPCE.

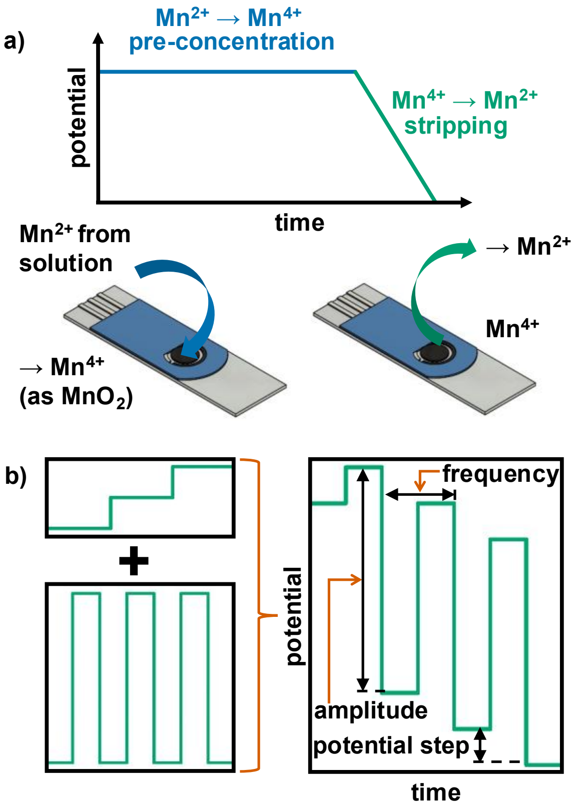

Figure S4. Schematic representation of square wave cathodic stripping voltammetry. **(a)** Pre-concentration of Mn(II) by oxidation to Mn(IV) and stripping via reduction of Mn(IV) back to Mn(II). **(b)** Combination of staircase and square waveforms to obtain the waveform used during the stripping step.

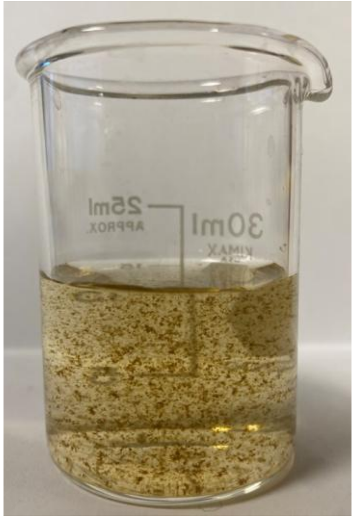

Figure S5. Brown precipitate formed after adding Mn(II) to acetate buffer at pH 11.8.

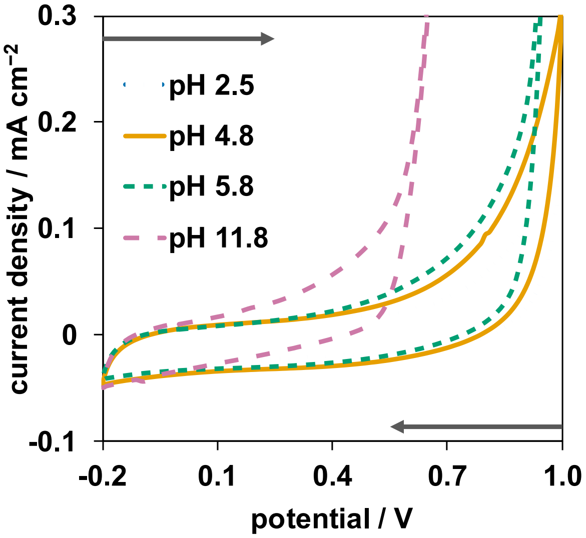

Figure S6. Cyclic voltammograms recorded at 0.1 V s^{-1} from −0.2 V to 1.0 V (vs Ag/AgCl pseudo-reference electrode) in a 0.1 M potassium acetate solution at pHs of 2.5 (blue), 4.8 (orange), 5.8 (green), and 11.8 (pink). The grey arrows indicate the directions of the measurements. Measurements were done using an unstirred batch setup.

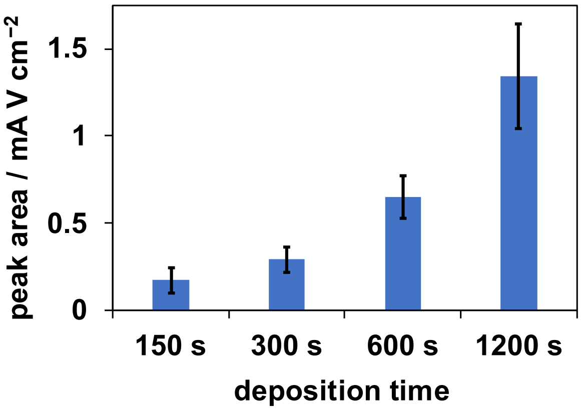

Figure S7. Change in peak areas of the obtained square wave voltammograms (stripping step from 0.8 V to 0 V with 25 mV of amplitude, 4 mV of potential step, and 14.24 Hz of frequency (vs Ag/AgCl pseudo-reference electrode)) from the analysis of 0.1 M potassium acetate solutions containing 100 µg L^{-1} Mn(II), with varying deposition times (150 s, 300 s, 600 s, and 1200 s) and a constant deposition potential of 0.9 V. Measurements were done with a stirred batch setup. The error bars represent standard deviations obtained from three replicates.

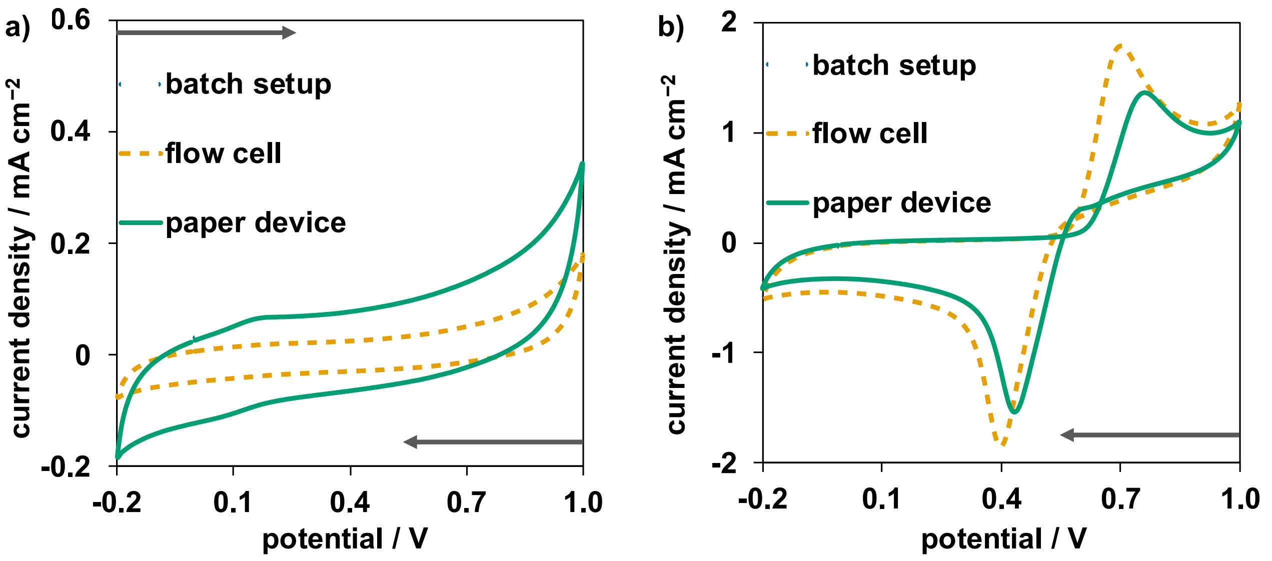

Figure S8. Cyclic voltammograms recorded for the batch (blue), flow cell (orange), and paper-based (green) setups, at 0.1 V s^{-1} from −0.2 V to 1.0 V (vs Ag/AgCl pseudo-reference electrode) in a 0.1 M potassium acetate solution at pH 4.8, in **(a)** the absence and **(b)** the presence of 10 mg L^{-1} Mn(II). The grey arrows indicate the directions of the measurements.

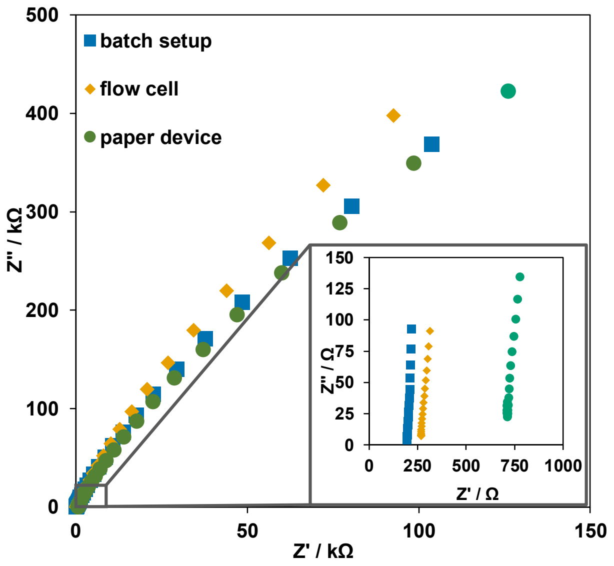

Figure S9. Impedance spectra obtained with the batch setup (blue), flow cell (orange), and paper device (green) using a 0.1 M potassium acetate solution at pH 4.8 containing 10 mg L^{-1} Mn(II). The spectra were recorded using a 0.1 V excitation amplitude in the frequency range of 100 kHz to 10 mHz. The inset figure shows the 1 kHz to 100 kHz frequency range.

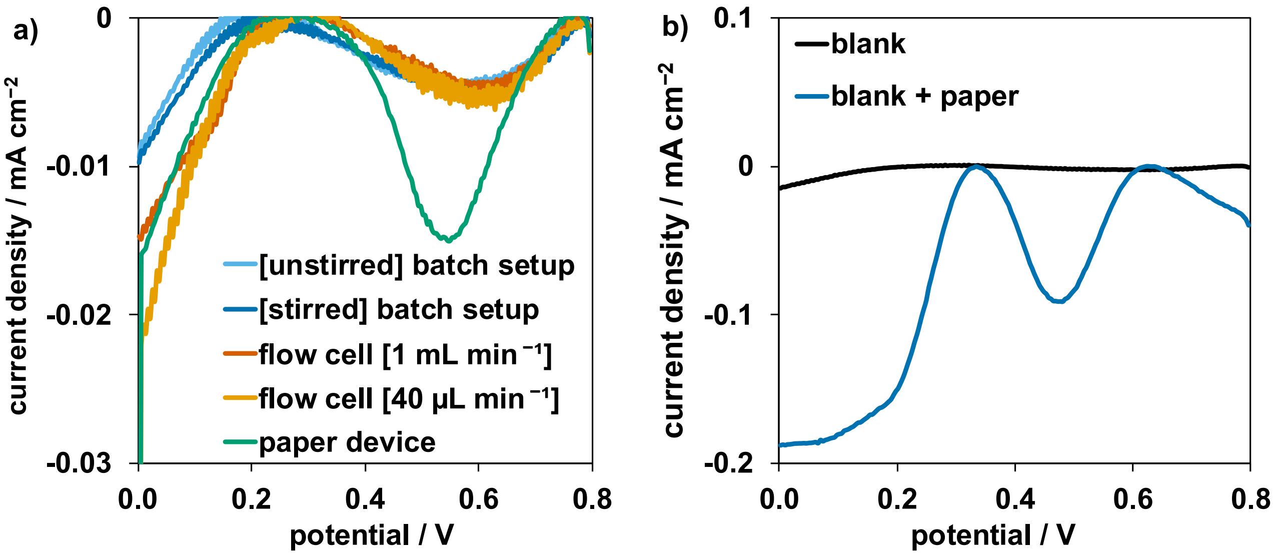

Figure S10. Blank square wave voltammograms of 0.1 M potassium acetate at pH 4.8 using different measurement conditions. **(a)** Square wave voltammograms using different measurement setups and flow conditions (indicated in parentheses). **(b)** Square wave voltammograms from measurements using the stirred batch setup with 0.1 M potassium acetate buffer (black line) and Whatman 4 filter paper dissolved in 0.1 M potassium acetate buffer (blue line).

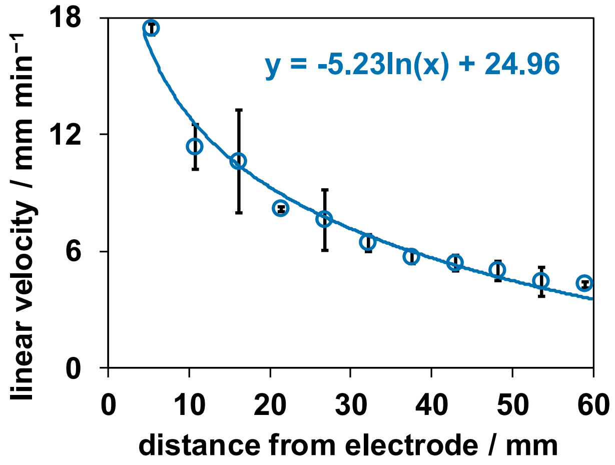

Figure S11. Flow profile of water (blue food dye added for visibility) flowing through the paper device with linear velocity calculated at intervals of 5.4 mm.

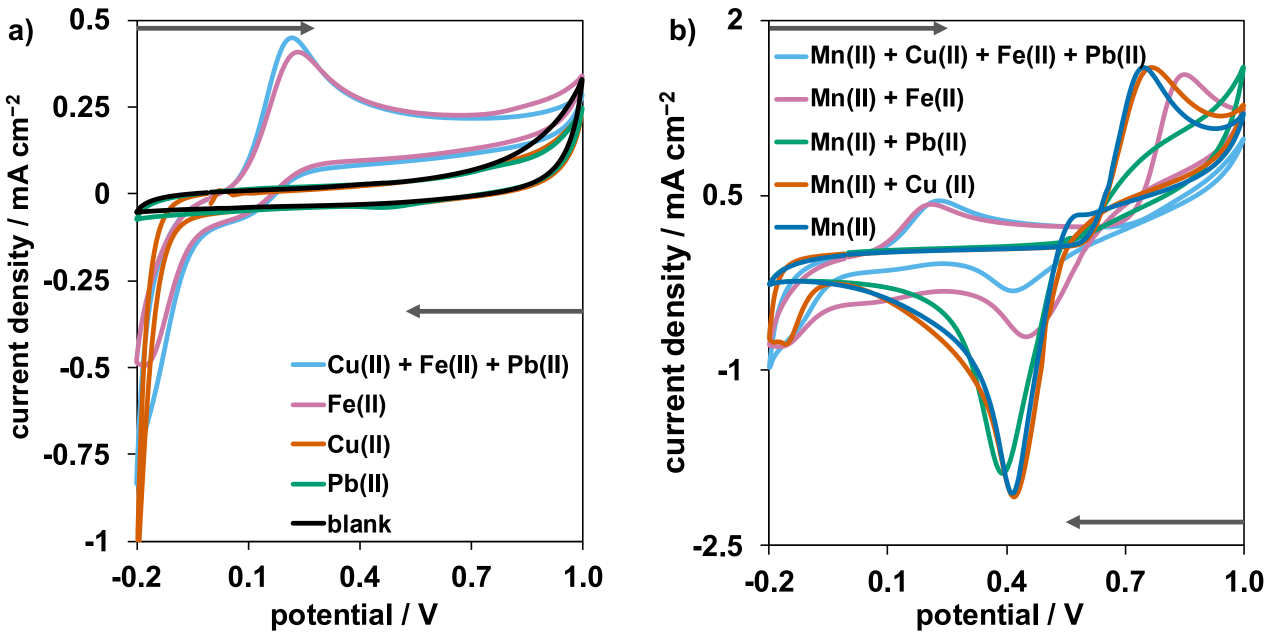

Figure S12. Cyclic voltammograms recorded at 0.1 V s^{-1} from −0.2 V to 1.0 V (vs Ag/AgCl pseudo-reference electrode) in a 0.1 M potassium acetate solution containing interferents and/or Mn(II). **(a)** Interferents present at 10 mg L^{-1}. **(b)** Mn(II) and interferents present at 10 mg L^{-1}. The grey arrows indicate the directions of the measurements.
